# Fluence Map Prediction Using Deep Learning Models – Direct Plan Generation for Pancreas Stereotactic Body Radiation Therapy

**DOI:** 10.3389/frai.2020.00068

**Published:** 2020-09-08

**Authors:** Wentao Wang, Yang Sheng, Chunhao Wang, Jiahan Zhang, Xinyi Li, Manisha Palta, Brian Czito, Christopher G. Willett, Qiuwen Wu, Yaorong Ge, Fang-Fang Yin, Q. Jackie Wu

**Affiliations:** ^1^Department of Radiation Oncology, Duke University Medical Center, Durham, NC, United States; ^2^Medical Physics Graduate Program, Duke University, Durham, NC, United States; ^3^Department of Software and Information Systems, University of North Carolina at Charlotte, Charlotte, NC, United States

**Keywords:** deep learning, artificial intelligence, fluence map, treatment planning, convolutional neural network, pancreas, SBRT

## Abstract

**Purpose:** Treatment planning for pancreas stereotactic body radiation therapy (SBRT) is a difficult and time-consuming task. In this study, we aim to develop a novel deep learning framework to generate clinical-quality plans by direct prediction of fluence maps from patient anatomy using convolutional neural networks (CNNs).

**Materials and Methods:** Our proposed framework utilizes two CNNs to predict intensity-modulated radiation therapy fluence maps and generate deliverable plans: (1) Field-dose CNN predicts field-dose distributions in the region of interest using planning images and structure contours; (2) a fluence map CNN predicts the final fluence map per beam using the predicted field dose projected onto the beam's eye view. The predicted fluence maps were subsequently imported into the treatment planning system for leaf sequencing and final dose calculation (model-predicted plans). One hundred patients previously treated with pancreas SBRT were included in this retrospective study, and they were split into 85 training cases and 15 test cases. For each network, 10% of training data were randomly selected for model validation. Nine-beam benchmark plans with standardized target prescription and organ-at-risk constraints were planned by experienced clinical physicists and used as the gold standard to train the model. Model-predicted plans were compared with benchmark plans in terms of dosimetric endpoints, fluence map deliverability, and total monitor units.

**Results:** The average time for fluence-map prediction per patient was 7.1 s. Comparing model-predicted plans with benchmark plans, target mean dose, maximum dose (0.1 cc), and D_95%_ absolute differences in percentages of prescription were 0.1, 3.9, and 2.1%, respectively; organ-at-risk mean dose and maximum dose (0.1 cc) absolute differences were 0.2 and 4.4%, respectively. The predicted plans had fluence map gamma indices (97.69 ± 0.96% vs. 98.14 ± 0.74%) and total monitor units (2,122 ± 281 vs. 2,265 ± 373) that were comparable to the benchmark plans.

**Conclusions:** We develop a novel deep learning framework for pancreas SBRT planning, which predicts a fluence map for each beam and can, therefore, bypass the lengthy inverse optimization process. The proposed framework could potentially change the paradigm of treatment planning by harnessing the power of deep learning to generate clinically deliverable plans in seconds.

## Introduction

Pancreatic cancer is an aggressive and lethal malignancy that accounted for an estimated 4.5% of all cancer-related deaths worldwide in 2018 (Bray et al., 2018). Stereotactic body radiation therapy (SBRT) utilizes sophisticated image-guidance and motion-management techniques to allow the delivery of a highly conformal dose of radiation to the target while sparing the surrounding normal tissues. Due to the nature of the higher fractional dose, achieving steeper dose gradients is prioritized to better spare the gastrointestinal (GI) organs at risk (OARs), such as the stomach and duodenum/small bowel. In addition, the highly variable planning target volume (PTV) and OAR geometry make the planning task extremely challenging. Although limiting the OAR maximum dose frequently outweighs target coverage, a trial-and-error process attempts to cover as much of the target with a prescription dose as possible. The consistency of plan quality is hard to maintain due to time pressure and the planner's experience, which may result in suboptimal plans. A system capable of maintaining consistently high plan quality is warranted in modern radiation oncology departments.

Over the last decade, efforts have been made to implement treatment-planning automation. Machine learning (ML) algorithms have been utilized to extract clinical knowledge from existing plans and apply it in various formats to create plans for new patients, which is known as knowledge-based planning (KBP). One KBP approach relies on patient-specific, dose-volume histogram (DVH) prediction to guide inverse optimization. Such modeling is based on the patient's anatomical structures and prior planning knowledge. Traditional ML techniques have seen significant success in DVH prediction for many treatment sites (Zhu et al., 2011; Yuan et al., 2012; Good et al., 2013; Skarpman Munter and Sjolund, 2015). Another approach is voxel-wise dose prediction–based treatment-planning guidance. Over the past several years, a shape-based method (Liu et al., 2015), atlas-selection methods (Sheng et al., 2015; McIntosh and Purdie, 2016, 2017), and artificial neural network methods using handcrafted features (Shiraishi and Moore, 2016; Campbell et al., 2017) were proposed. Recently, convolutional neural networks (CNNs) have shown success in predicting 3-D dose distributions (Kearney et al., 2018; Barragán-Montero et al., 2019; Chen et al., 2019; Fan et al., 2019; Nguyen et al., 2019a,b). This type of model is typically referred to as a deep learning (DL) model. A majority of these models employ network structures similar to U-Net, which was initially developed for biomedical image segmentation (Ronneberger et al., 2015). However, in this approach, a second step of plan generation via inverse optimization is necessary to create a treatment plan aiming to achieve the predictions (McIntosh et al., 2017; Fan et al., 2019), either as DVH-based optimization or as voxel-based dose mimicking.

We contend that high-quality radiotherapy plans with standardized dose constraints and beam settings can be directly created by predicting their fluence maps without optimization or dose mimicking. We refer to this process as direct plan generation (as opposed to the automated planning process used in the literature that generally requires two steps as mentioned above). Few publications have focused on direct fluence map prediction (Lee et al., 2019; Sheng et al., 2019). In the case of whole breast irradiation, fluence prediction was achieved with a random forest model proposed by Sheng et al. (2019). Lee et al. (2019) show that, given the organ contours and the complete set of field-dose distributions, fluence maps for seven-beam prostate IMRT could be reconstructed by a modified U-Net with high accuracy. However, the study did not investigate how to obtain the known field dose. Rather, the authors assumed the field doses were a prerequisite for their technique to work. Indeed, solving the field dose of each beam remains a challenge. We hypothesize that anatomical planning features, together with the physician's planning objectives, could lead to accurate prediction of the field doses of each beam and their corresponding fluence maps. The expert planner incorporates the physician's planning objectives during manual planning. Therefore, these planning objectives are embedded in these plans, and DL models should be able to capture such information in the training data. In this feasibility study, we present a novel deep learning framework for direct fluence map prediction (a.k.a. direct plan generation) and demonstrate its performance using clinical pancreas SBRT cases.

## Materials and Methods

### Patient Selection and Radiation Therapy Plan

One hundred pancreatic cancer patients previously treated with SBRT at Duke University Medical Center between 2014 and 2019 were included in this retrospective study. This study was approved by the institutional review board. In clinical plans, the dose prescription to the PTV was 25 Gy, often with a simultaneous integrated boost to the internal gross tumor volume (iGTV) with 33 or 40 Gy. The GI OAR (stomach, C-loop/duodenum, and bowels) dose constraints varied in maximum dose and maximum volume according to the different physician preferences. We aim to develop a model that is capable of generating clinical-quality pancreas SBRT IMRT plans. In this feasibility study, each case was replanned by experienced clinical physicists who specialized in GI SBRT using unified planning objectives and a standardized IMRT protocol with a single prescription level. The prescription for both the PTV and iGTV were 33 Gy in five fractions. All plans were designed with nine equally spaced coplanar 10-MV photon beams. Stomach, C-loop/duodenum, and bowels were combined and referred to as the OAR. The maximum dose for the OAR was limited to 25 Gy (0.1 cc). This protocol creates the scenario of an inverted relationship of target and OAR dose prescription, a clinical scenario that often has to be handled manually by an experienced planner for each case. In the following, we refer to the resulting standardized plans as the benchmark plans, which were used to train the model. The same beam orientations, including gantry angles, and beam shape definition via its open field dose, referred to as beam templates, were also included as input for the DL models. The 100 patient cases were divided randomly into an 85:15 training:testing ratio. All treatment plans were generated in the Eclipse® Treatment Planning System (TPS) (Varian Medical Systems, Palo Alto, CA) version 13.7 with the volume dose calculated by the Analytical Anisotropic Algorithm version 13.7.14. A Varian Millennium 120 multi-leaf collimator (MLC) was used to deliver the modulated fluence maps. The leaf-sequencing algorithm used was Smart LMC version 13.7.14.

### Study Workflow

**Figure 1:**
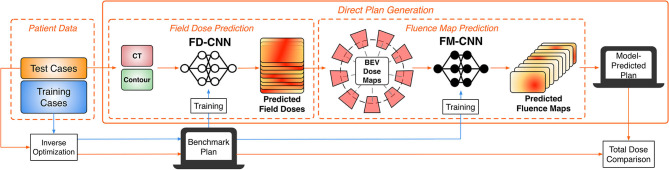
Overall workflow of the DL modeling and validation. The prediction pipeline generates fluence maps from CT data and structure contours.

The overall study workflow is summarized in Figure 1. The proposed framework adopts a pipeline structure, in which two CNNs make consecutive predictions to generate the complete plan with fluence maps. The input into the pipeline includes planning computed tomography (CT) images as well as contours of the PTV and OARs. First, the field-dose CNN (FD-CNN) predicts 9 individual IMRT field dose distributions, i.e., FD-CNN field dose from CT and structure contours. Next, each 3-D field dose is projected along the beam's eye view (BEV), generating the 2-D BEV dose map. Finally, the fluence map CNN (FM-CNN) predicts the fluence map for each beam from the corresponding BEV dose map. The two CNNs were implemented in Keras with the Tensorflow backend and trained separately. The entire model was trained on a workstation with an Intel Xeon E5 v4 processor, 64 GB of RAM, and an NVIDIA Quadro M4000 graphics card. In order to evaluate the proposed framework's performance, we compared the automatically generated plans using the DL technique described in this research study, referred to as “model-predicted plans,” against the benchmark plans generated by human experts using the standard inverse planning process.

#### Data Preprocessing

All plans, including CT images, contours, field doses, and fluence maps, were exported from the Eclipse TPS as DICOM files. As the original plans have different spatial resolutions, resampling was performed on dose and contour images with 1 mm axial resolution and 2 mm slice thickness. Linear interpolation was used to increase the resolution of dose distributions to facilitate more accurate dose prediction. Relative values were used in field doses with the prescription dose of 33 Gy normalized to 100%. Axial slices were cropped to a 192 × 192 pixel image centered at the isocenter. Fluence maps and other BEV projections had a resolution of 2.5 × 2.5 mm^2^ at the isocenter plane. All the training data were randomly shuffled before holding out a validation set.

#### Field Dose Prediction

**Figure 2:**
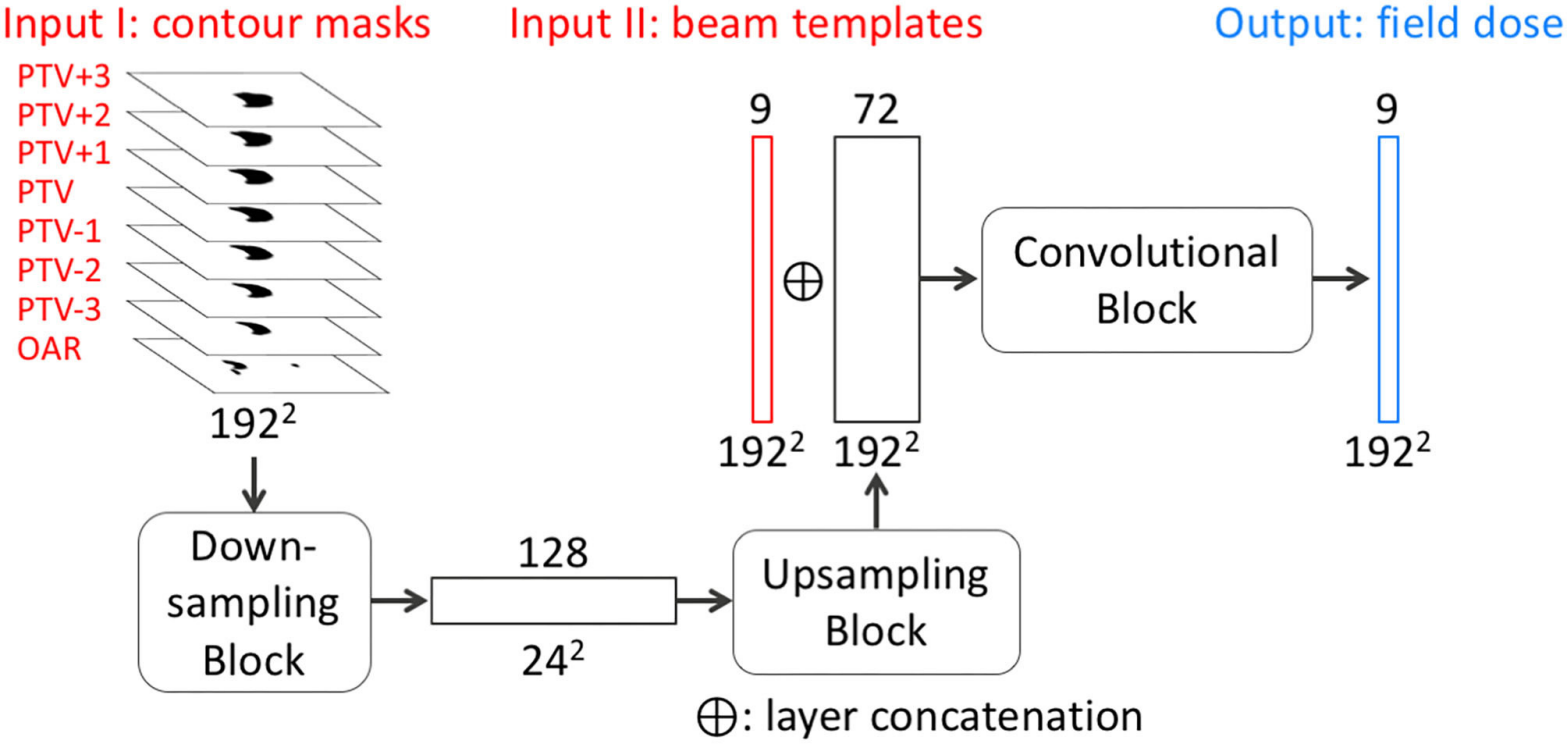
Simplified network architecture of FD-CNN. FD-CNN takes contour masks and beam templates as input and predicts nine field doses in an axial slice. The details of downsampling, upsampling, and convolutional blocks (rounded rectangles) are omitted to highlight the transformation process from the inputs to the output. *PTV*±*n* refers to the n th PTV slice superior or inferior to the query slice. *OAR* includes only the OAR contour in the query slice. Each rectangle block represents a layer with the number of channels on the top and image dimensions labeled on the bottom of each layer.

The objective of FD-CNN is to predict field doses from CT images and structure contours. The network architecture of FD-CNN is illustrated in Figure 2, and it operates on a slice-by-slice basis. To predict field dose in one query slice, the main input includes seven PTV slices (the query slice and six adjacent slices) and the OAR query slice, which are all 192 × 192 binary masks. The adjacent PTV slices were included to account for PTV shape change in the superior–inferior direction. In the downsampling block, the contour masks were downsampled three times using strided 2-D convolution to produce 128 channels of 24 × 24 feature images. An upsampling block produced 72-channel feature images, using strided 2-D transposed convolution three times to restore the 192 × 192 resolution. CT images were incorporated in the form of beam templates (Input II in Figure 2) calculated by the TPS and concatenated to the 72-channel feature images. A final convolution block was applied to produce nine field doses for the nine equally spaced beams. The prediction region was limited to a region of interest (ROI), which was the PTV expanded by 1 cm. The Swish activation function (Ramachandran et al., 2017) was used in the network to introduce non-linearity. Swish is the product of an identity function and a sigmoid function, which can be expressed as

(1) $$\begin{array}{r} Swish\left(x\right)=\frac{x}{e^{-x}+1}. \end{array}$$

In predicting all field doses, the total dose was acquired automatically by summation. The loss function of FD-CNN (***L***_***FD***_) was the sum of two parts: field dose (FD) error and total dose (TD) error in the ROI, which is formulated as

(2) $$\begin{array}{l} L_{FD}=\frac{1}{N\left(ROI\right)}\\ \left[\sum_{beam}\sum_{ROI}{\left(FD_{bench}-FD_{pred}\right)}^{2}+\mu .\sum_{ROI}{\left(TD_{bench}-TD_{pred}\right)}^{2}\right] \end{array}$$

***N***(***ROI***) is the number of ROI pixels. ***FD***_***bench***_ and ***TD***_***bench***_ are the benchmark plan field and total doses. ***FD***_***pred***_ and ***TD***_***pred***_ are the predicted field and total doses. The field and total dose error terms were summed with the regularization term of μ as tuned by validation. All slices with ROI were used to predict field dose by FD-CNN. For each patient, all the predicted 2-D dose slices were stacked together to form the predicted 3-D dose distributions of a given beam. In total, there were 3,238 slices from all 85 training cases. The benchmark plan's field dose is used as the ground truth for model training. Ten percent of the training slices were held out for validation. FD-CNN was trained using an Adam optimizer (Kingma and Ba, 2014) with a learning rate of 0.001 and early stopping with patience of eight epochs (training terminates when validation loss does not improve for eight epochs).

#### Fluence Map Prediction

**Figure 3:**
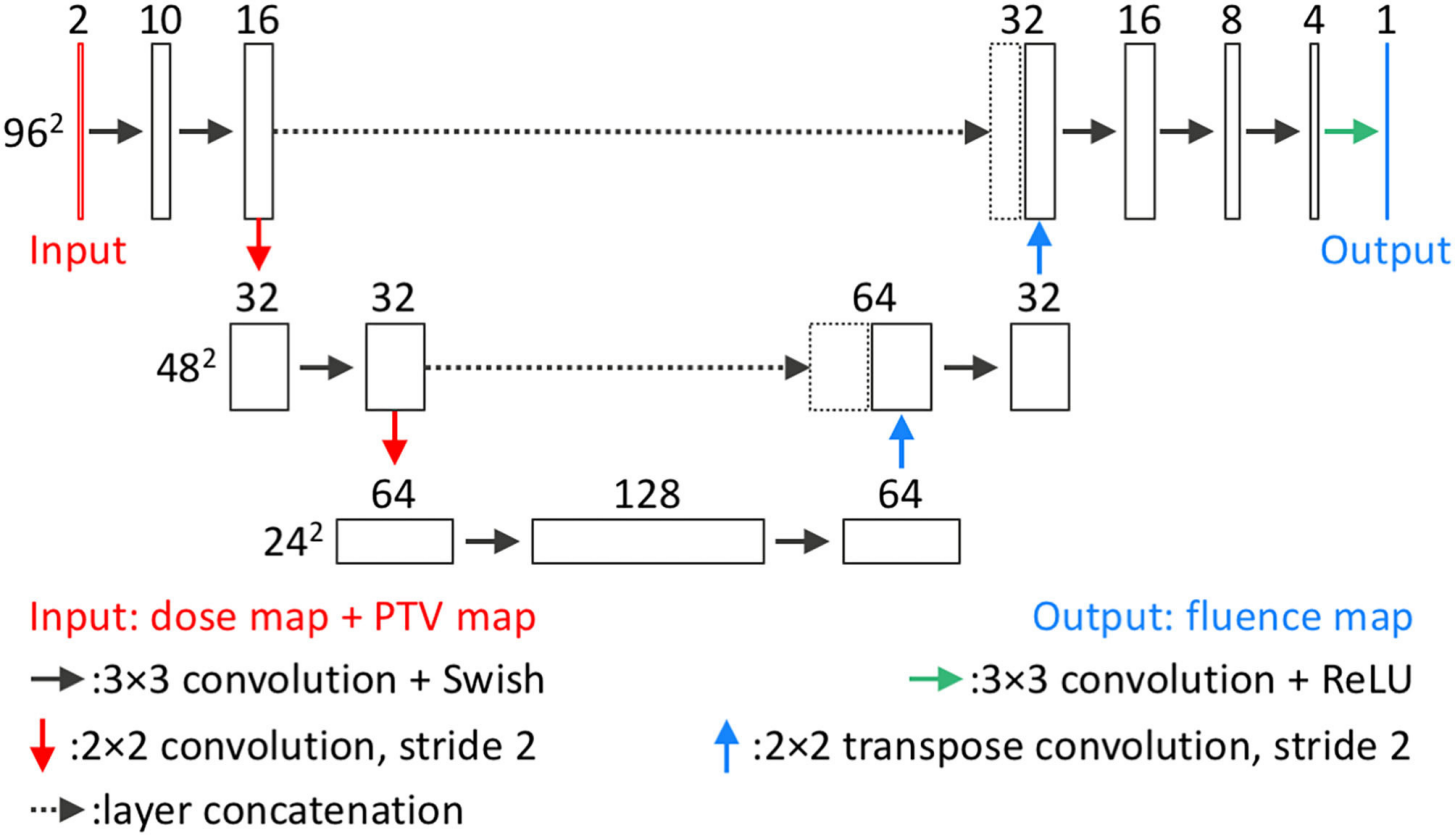
Network architecture of FM-CNN. For each beam, FM-CNN predicts the fluence map from the dose map and PTV map (concatenated). Three hierarchies of image dimension (96, 48, 24 pixels) are used. Each rectangular block represents a layer with the number of channels on the top and image dimensions labeled on the left of each hierarchy.

The second DL model is the FM-CNN, which predicts one fluence map from each 3-D field dose. The network architecture of FM-CNN is illustrated in Figure 3. It adopts a customized U-Net shape, which includes three resolution hierarchies (96, 48, and 24 pixels). The inputs of FM-CNN are the BEV dose map and the BEV PTV map, and the output is the fluence map. For one beam, the BEV dose map is the projection of the predicted field dose along the BEV, and the BEV PTV map is the binary projection of the PTV contour along the BEV. The upsampling and downsampling were achieved with strided 2-D convolution and strided 2-D transposed convolution, respectively. The BEV dose maps and fluence maps of the benchmark plans serve as ground truth for model training. The loss function of FM-CNN (***L***_***FM***_) is a modified mean absolute error (MAE), which is formulated as

(3) $$\begin{array}{r} L_{FM}=\left(1+\lambda \right)\frac{\sum |y_{bench}-y_{pred}|}{N\left(y_{bench}>0\right)} \end{array}$$

where ***y***_***bench***_ and ***y***_***pred***_ are the benchmark and predicted values of the fluence map, and ***N*(*y***_***bench***_** > 0)** is the count of benchmark fluence map pixels with non-zero values. The factor **λ** is the regularization term to prevent FM-CNN from over- or underestimating the fluence maps overall. It is expressed as

(4) $$\begin{array}{r} \lambda =\\ \frac{\left|N\left(y_{bench}-y_{pred}>0.001\right)-N\left(y_{bench}-y_{pred}<-\;0.001\right)\right|}{N\left(y_{bench}>0\right)} \end{array}$$

Because fluence intensity is directly linked to field dose, the fluence prediction error should have a mean value close to zero in order to avoid overdosing and underdosing. Therefore, this regularization factor is added to control the mean value of prediction error for all pixels and keep the numbers of positive and negative errors at the same level.

The total training data size was 765 for 85 patients, of which 10% were held out for validation. The model was trained using an Adam optimizer with a learning rate of 0.001 and early stopping with patience of 15 epochs.

In the final validation step, these predicted fluence maps were subsequently imported into the TPS for leaf sequencing and dose calculation. The resulting plans are referred as model-predicted plans and are compared to the benchmark plans for overall performance.

### Model Assessment

For model evaluation, the benchmark plan is considered as the ground truth. Each of the two models is evaluated separately and then collectively for dosimetric quality and deliverability. The FD-CNN field dose is compared with the corresponding field dose of the benchmark plan to evaluate FD-CNN performance. To evaluate FM-CNN performance, a special plan, the FM-CNN plan, is generated by FM-CNN using the field dose from the benchmark plan, thus eliminating error contamination from the first CNN model. The model-predicted plan is the final plan created with the fluence map predicted by the complete model (i.e., both CNNs) and, thus, evaluates the overall performance of the framework.

The 15 cases not included in model training were used as an independent test set, which consists of 638 slices and 135 fluence maps. For each test case, an FD-CNN field dose, an FM-CNN plan, and a model-predicted plan were created. The voxel-wise percentage dose difference Δ***D*** is calculated as

(5) $$\begin{array}{l} \Delta D(V)=\frac{1}{N(V)}\sum_{i\in V}\left|\frac{D_{bench}^{\left(i\right)}-D_{pred}^{\left(i\right)}}{D_{prescription}}\right|\times 100 \end{array}$$

***V*** is the calculation volume, and ***N*(*V*)** is the number of voxels in this volume. Several dosimetric endpoints were also used for assessment. These include PTV max dose (0.1 cc), mean dose, D_95%_ for the PTV, and mean and max doses (0.1 cc) for the OARs. To provide a direct assessment of fluence map prediction, MAEs were calculated between FM-CNN and benchmark fluence maps.

In Eclipse TPS, optimal fluence maps, generated by inverse optimization or deep learning models, are converted to actual fluence maps by leaf-sequencing algorithms to enable delivery on the machine. Unrealistic optimal fluence map features, such as extremely heterogeneous regions or high transmission value at a single pixel, could potentially result in a large discrepancy between optimized and delivered doses. Therefore, the deliverability of fluence maps was measured by the gamma index between optimal (before leaf sequencing) and actual fluence maps (after leaf sequencing) for both benchmark and predicted plans. We employed the gamma analysis in a similar fashion and intent as IMRT quality assurance. Here, a high gamma passing rate indicates that the optimal fluence map is physically realistic and could be achieved by the leaf-sequencing algorithm. Gamma analysis was performed using an in-house program with a 3%/3 mm criterion. Total monitor units (MUs) from benchmark and model-predicted plans were compared.

After the DL framework was completely trained and tested, we reduced the training cases for both CNNs and calculated the loss values on the test set. In addition, a series of ablation studies were conducted, in which certain CNN components were removed to test the model performance. For the FD-CNN model, we removed the input of one, two, or three pairs of adjacent PTV slices or beam templates. For the FM-CNN model, we removed the input of the PTV map. The reduced models were evaluated on the same test set and compared with the original models.

## Results

### Model Training

The model training details are summarized in Table 1. FD-CNN has 3.35 million trainable parameters and took 3 h to train. FM-CNN has a much less complex architecture with 0.20 million trainable parameters and took 4 min to train. The projection of field dose and PTV along the BEV is relatively time-consuming compared to CNN predictions. On average, prediction of nine fluence maps for each patient took 7.1 s, including 1.10 s for FD-CNN prediction, 5.97 s for BEV projection, and 0.03 s for FM-CNN prediction. In the entire workflow, the computation time of the model is typically less than that of TPS dose calculation. A DL model-predicted plan was generated within 1 to 2 min, including calculating the model-predicted plan dose in TPS, as compared to the traditional manual planning, which takes between 1 and 3 h.

**Table 1:** Model training and calculation details.

	**Trainable parameters**	**Training data size**	**Epochs**	**Training time**	**Calculation time per image**	**Calculation time per patient**
FD-CNN	3,351,185	3,238	48	3 h	0.026 s	1.100 s
BEV projection	n/a	n/a	n/a	n/a	0.663 s	5.966 s
FM-CNN	203,621	765	134	4 min	0.003 s	0.030 s

*The training details of two CNNs include the number of trainable parameters, training epochs, and training time. BEV projection is a deterministic process that requires no training. The calculation times listed are average prediction time of CNNs and average calculation times of BEV projection*.

### Model Assessment

**Figure 4:**
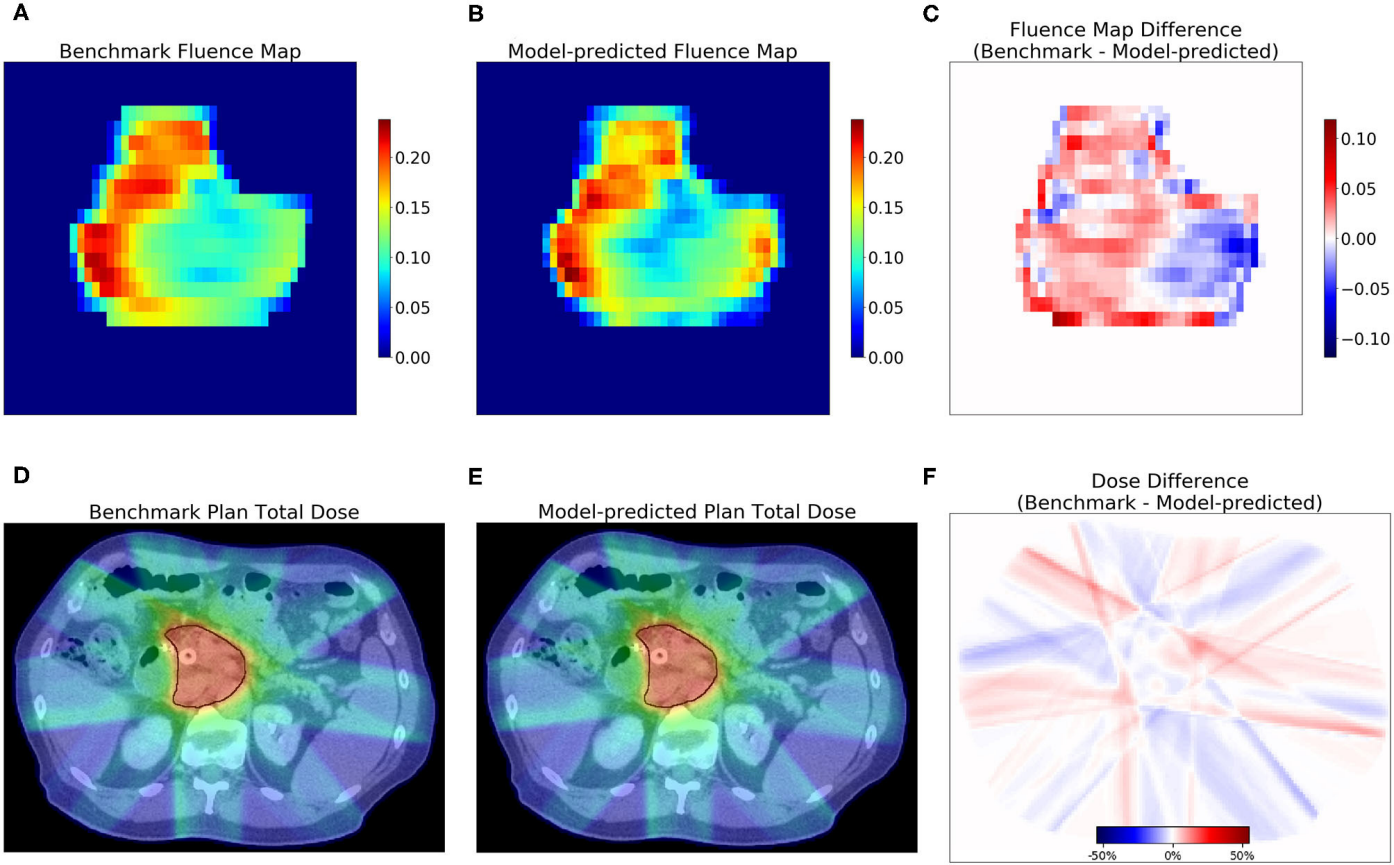
Examples of fluence map and dose comparisons with the benchmark in one test case. The model-predicted fluence map recreated the fluence contrast in the benchmark. The model-predicted plan achieved a similar total dose as the benchmark. The first row shows the benchmark **(A)** and model-predicted **(B)** fluence maps of one beam, and the difference **(C)**. The second row shows one axial slice of the dose distribution of benchmark plan **(D)** and model-predicted plan **(E)** and the dose difference **(F)**. PTV contour is marked with black lines in **(D,E)**.

**Figure 5:**
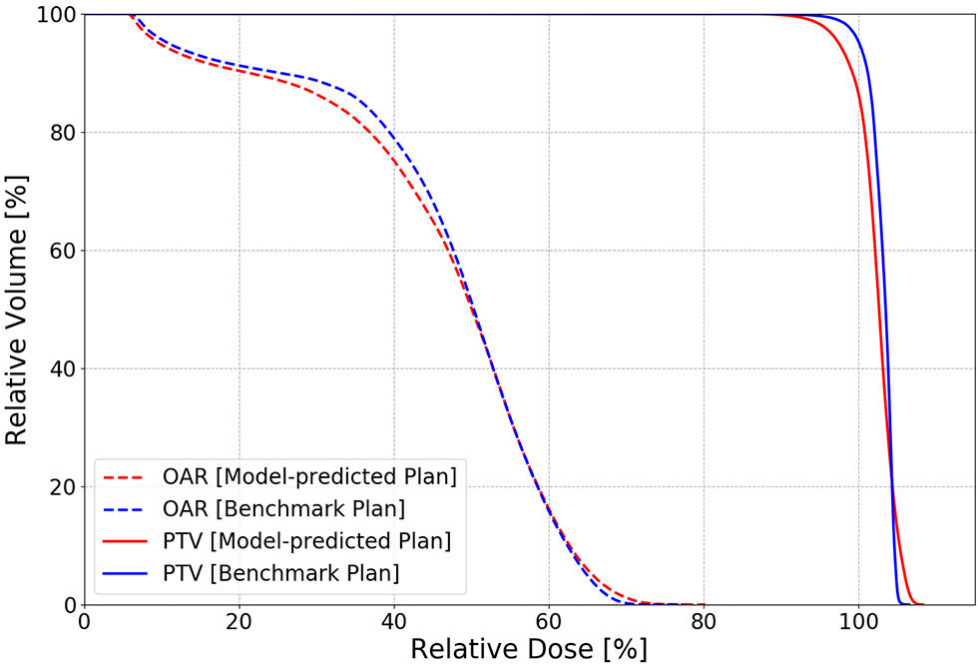
An example of PTV (solid) and OAR (dashed) DVH comparison in one test case between the benchmark (blue) and model-predicted (red) plans. The benchmark plan has slightly better PTV homogeneity than the model-predicted plan with the FM-CNN plan in between.

**Figure 6:**
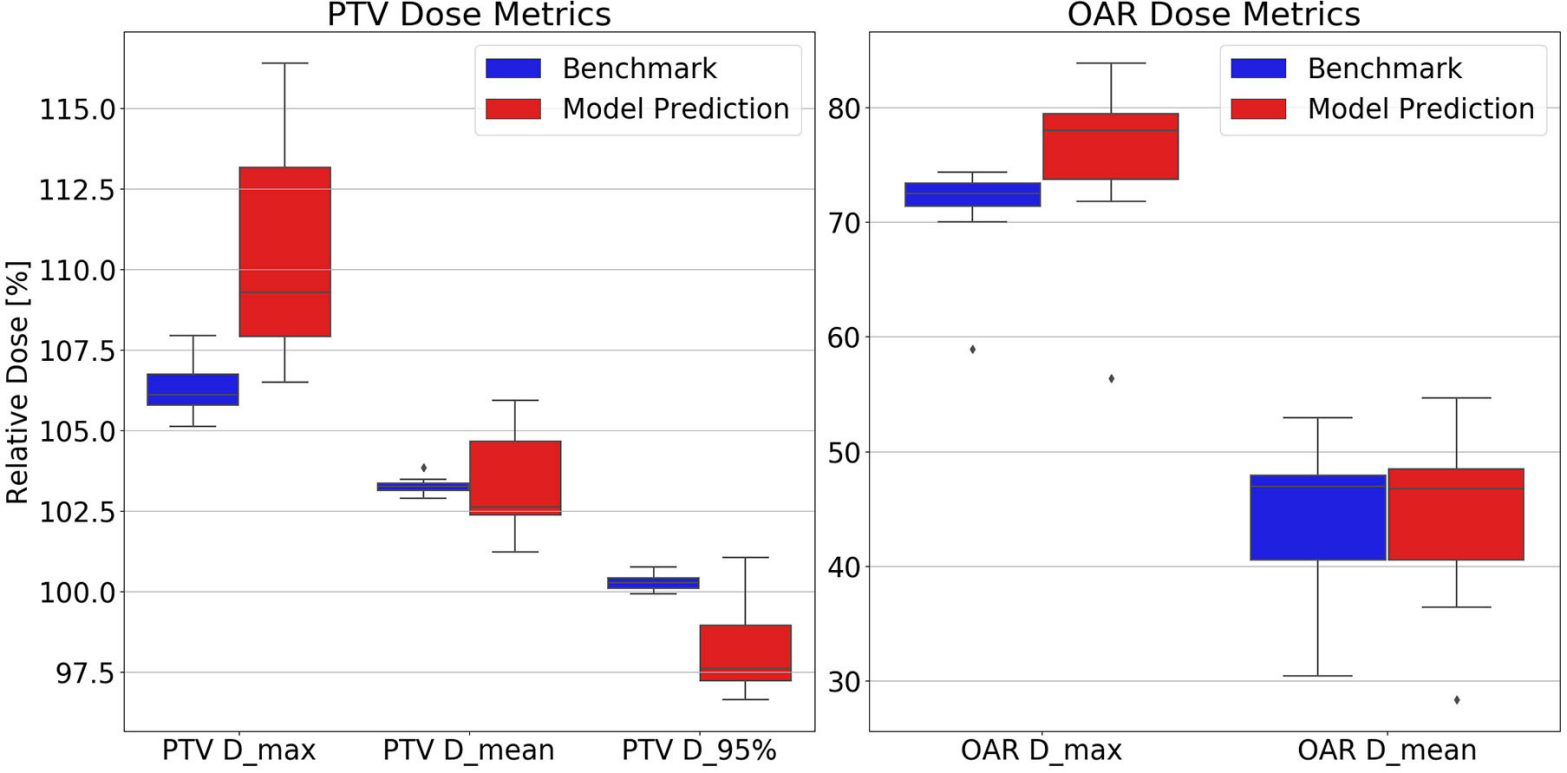
Test set distributions of PTV **(Left)** and OAR **(Right)** dose metrics comparing benchmark and model-predicted plans. Model-predicted plans have higher PTV and OAR maximum dose and lower D_95%_ than the other plan groups. Dose values are reported as percentage of the prescription dose. D_max_, maximum dose; D_mean_, mean dose; D_95%_, minimum dose received by 95% of the volume.

The dosimetric evaluation results are summarized in Table 2. Here, the ground truth is the dose from the benchmark plans. Model-predicted plans have the largest dose differences among the three evaluation plans, and they represent the overall performance of the workflow. In the deliverable plans, i.e., FM-CNN and model-predicted plans, PTV and OAR (stomach, C-loop/duodenum, and bowels combined) maximum dose errors are larger than mean dose errors. Figure 4 compares the total dose distribution between the model-predicted and benchmark plans of an example case. Figure 5 compares the DVH for the same case. As shown in the figure, the predicted fluence map achieves similar fluence modulation as the fluence map of the benchmark plan. Further, the TPS-calculated dose distribution of the predicted plan exhibits small differences from the corresponding benchmark plan, indicating highly similar plan quality. The distributions of PTV and OAR dose metrics of benchmark and model-predicted plans are plotted in Figure 6.

**Table 2:** Dose differences between all predicted plan groups and benchmark plans.

**Plan type**	**Dose type**	**Region**	**Voxel dose difference [%]**	**D_**mean**_ difference [%]**	**D_**max**_ difference [%]**
FD-CNN dose	Total dose (CNN)	ROI	1.79 ± 2.21	0.41 ± 0.28	0.48 ± 0.31
		PTV	0.91 ± 0.79	0.57 ± 0.25	0.48 ± 0.31
		ROI–PTV	2.65 ± 2.75	0.51 ± 0.34	0.49 ± 0.54
	Field dose (CNN)	ROI	1.25 ± 1.11	0.51 ± 0.42	1.82 ± 1.44
FM-CNN plan	Total dose (TPS)	PTV	1.22 ± 0.96	0.88 ± 0.65	1.46 ± 1.19
		OAR	0.86 ± 0.75	0.30 ± 0.17	0.86 ± 0.52
Model-predicted plan	Total dose (TPS)	PTV	2.41 ± 1.87	1.24 ± 0.74	4.10 ± 2.35
		OAR	2.70 ± 2.45	0.94 ± 0.65	4.77 ± 2.84

*Model-predicted plans exhibit larger dose differences than FD-CNN doses and FM-CNN plans*.

In terms of fluence map deliverability, the average ± standard deviation gamma passing rate was 98.14% ± 0.74% for benchmark plans and 97.69% ± 0.96% for model-predicted plans, respectively, which demonstrates highly similar deliverability. The average ± standard deviation of total MU per patient is 2,122 ± 281 in model-predicted plans and 2,265 ± 373 in benchmark plans.

**Figure 7:**
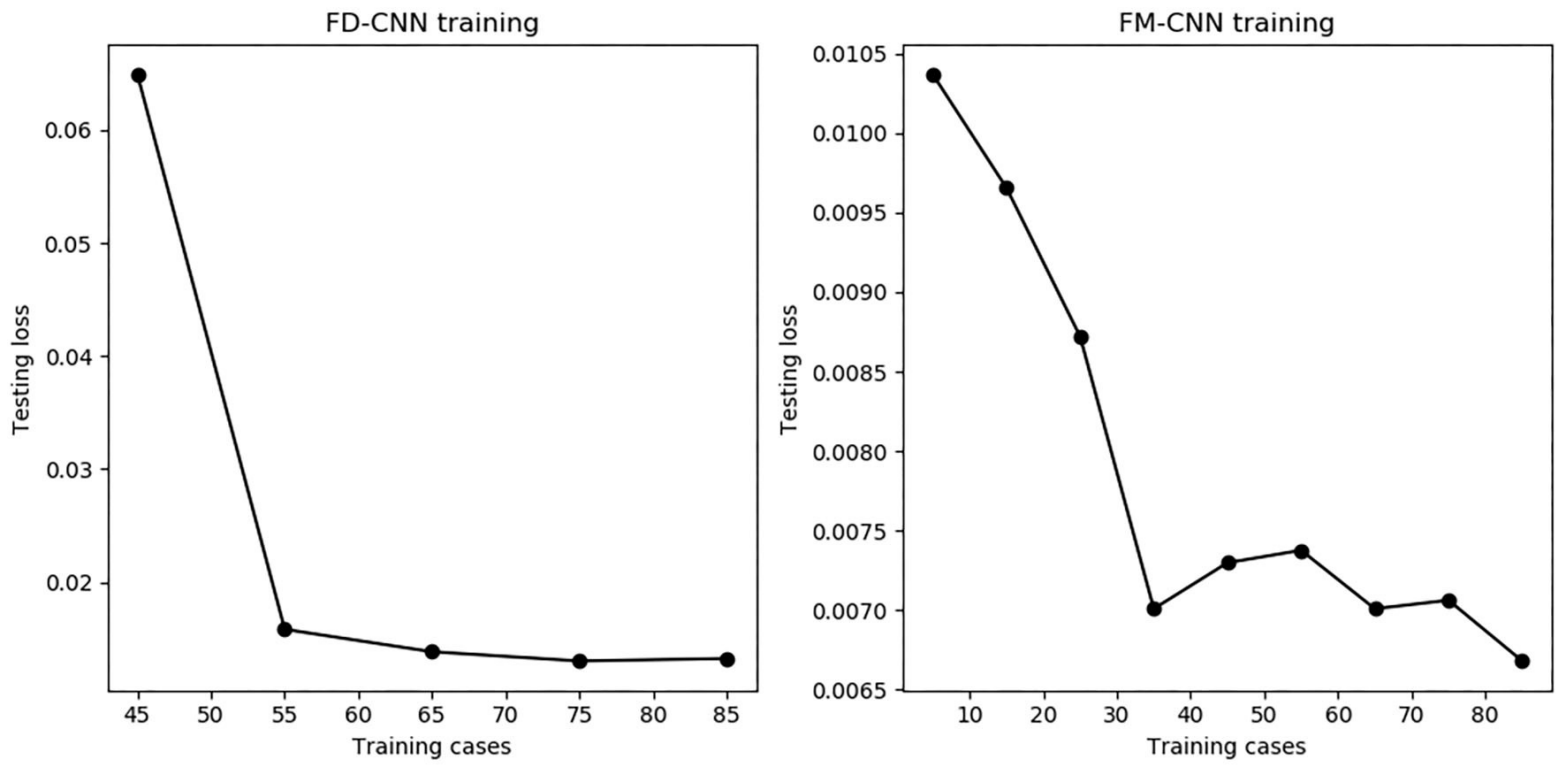
The number of training cases vs. testing loss for both CNNs. The testing loss stabilized when using 55 or more training cases for FD-CNN **(Left)** and 35 or more cases for FM-CNN **(Right)**.

The model performance of FD-CNN and FM-CNN were plotted against the number of training cases used, as shown in Figure 7. It can be seen that the testing loss of FD-CNN plateaued after 55 cases although FM-CNN required only 35 cases to achieve reasonably good performance. The ablation study showed that, for FD-CNN, removing the beam template input would increase the testing loss by 20%; using only four, two, and zero adjacent PTV slices would increase the testing loss by 7, 25, and 66%, respectively. For FM-CNN, removing the PTV map input would only slightly increase the testing loss by 1%.

## Discussion

We develop a novel deep learning framework to generate clinical-quality pancreas SBRT plans in seconds. It offers the advantage of bypassing lengthy optimization, during which the planner needs to adjust optimization objectives and aims to achieve similar performance as the human expert exercising inverse optimization. This study demonstrates the novel approach of AI-driven treatment planning via predicting fluence maps, thus providing a more complete approach to generating deliverable high-quality plans, which has not been sufficiently addressed in previous studies (Liu et al., 2015; Skarpman Munter and Sjolund, 2015; Kearney et al., 2018; Barragán-Montero et al., 2019; Chen et al., 2019; Nguyen et al., 2019a,b). Translating predictions from previous KBP models, either DVH-based or 3-D dose guidance, to the final deliverable plan has been challenging and remains a key implementation bottleneck in clinics. Efforts have been made to complement KBP models to arrive at the final plan (McIntosh et al., 2017; Long et al., 2018; Fan et al., 2019). The aim of the proposed DL solution is to garner knowledge from existing plans and generate deliverable plans for new patients, which falls under the broad KBP vision. Our approach directly predicts fluence maps rather than predicting achievable DVH/doses in other KBP approaches. More specifically, we use CNNs to establish the correlation between patient anatomy patterns and each individual beam's dose/fluence map, which has not been investigated in previous KBP studies. This approach is built upon beamlet-based fluence optimization, with which a subsequent leaf-sequencing process converts the fluence maps to MLC motion parameters. By replacing the FM-CNN, the proposed approach could also be employed along with direct aperture optimization (Shepard et al., 2002) in step-and-shoot IMRT, which would offer the advantage of fewer segments and MUs. This is an area of potential study that warrants future effort. It would also be of interest to compare the proposed approach with other KBP-based plan-generation methods in future studies.

We redesigned the entire radiation therapy treatment-planning workflow by incorporating DL models for dose prediction and fluence map generation, thereby completing AI-driven plan generation in seconds. Our results demonstrate that such AI-driven plans have similar quality when compared to manually generated inversely optimized plans although, more importantly, a ready-to-deliver plan is generated with no further human intervention needed. In dose prediction, the total dose in PTV predicted by FD-CNN achieved a similar level of accuracy as existing deep learning–based dose-prediction models. The input of adjacent PTV slices provided superior–inferior contour change information efficiently, which significantly reduced the testing loss while maintaining lower memory consumption than 3-D networks. The second step of our framework, i.e., fluence map prediction from an existing field dose, directly converts an individual field dose into its corresponding fluence map, which eliminates interplay among beams and require no optimization or intermediate dose calculation. With the existing ground truth field dose, we achieved similar fluence map MAE (mean value: 2.06 × 10^−3^) as Lee et al. (2019) (median value: 9.95 × 10^−4^). With similar fluence map prediction accuracy, our proposed framework is capable of directly predicting a fluence map from contour and CT alone, which Lee et al. (2019) has yet to achieve.

We used standardized nine-beam IMRT plans as a benchmark in this study, and this increased consistency in plan quality and reduced the need for a large amount of training data. We argue that the training data meticulously generated by human experts is optimal in terms of the endpoints of target coverage and luminal structure maximum dose. One limitation of the model is that the training and testing cases must have the same beam arrangement, dose prescription level, and physician preferences. Substantially more training data are anticipated to be required to train a model that incorporates different beam arrangements and dose constraints. This study focuses on pancreas SBRT although we are modifying and testing the model for other disease sites. With the current model, we do not think it is generically applicable to other disease sites. We anticipate that data from each specific site are required to train a robust model. Further study is underway to address these challenges.

## Conclusion

We develop a deep learning framework utilizing two CNNs to directly generate a clinical-quality IMRT plan from CT images and contours for pancreas SBRT. This framework changes the traditional approach of inverse treatment planning by replacing the inverse optimization engine with the intelligent neural networks. The proposed method has great potential to improve clinical efficiency and plan quality consistency for challenging treatment sites.

## Data Availability Statement

The raw data supporting the conclusions of this article will be made available by the authors, without undue reservation.

## Ethics Statement

The studies involving human participants were reviewed and approved by Duke University Health System Institutional Review Board. Written informed consent for participation was not required for this study in accordance with the national legislation and the institutional requirements.

## Author Contributions

WW and YS performed model development and plan comparison. CW and JZ performed patient selection and benchmark plan generation. MP, BC, and CW are GI physicians from whom we learned expert domain knowledge. QW, F-FY, and YG reviewed experiment design, paper content, and statistical analysis. QJW supervised the entire study and revised this paper. All authors contributed to the article and approved the submitted version.

### Conflict of Interest

The authors declare that the research was conducted in the absence of any commercial or financial relationships that could be construed as a potential conflict of interest.

## Abbreviations

BEV: beam's eye view
CNN: convolutional neural network
CT: computed tomography
DAO: direct aperture optimization
DL: deep learning
DVH: dose-volume histogram
FD: field dose
FM: fluence map
GI: gastrointestinal
iGTV: internal gross tumor volume
KBP: knowledge-based planning
MAE: mean absolute error
MU: monitor unit
OAR: organ-at-risk
PTV: planning target volume
ROI: region of interest
SBRT: stereotactic body radiation therapy
TPS: treatment planning system.
